# Dietary Patterns and Weight Status in Italian Preschoolers with Autism Spectrum Disorder and Typically Developing Children

**DOI:** 10.3390/nu13114039

**Published:** 2021-11-12

**Authors:** Benedetta Raspini, Margherita Prosperi, Letizia Guiducci, Elisa Santocchi, Raffaella Tancredi, Sara Calderoni, Maria Aurora Morales, Mariangela Morelli, Meg Simione, Lauren Fiechtner, Filippo Muratori, Hellas Cena

**Affiliations:** 1 Department of Public Health, Neurosciences, Experimental and Forensic Medicine, Section of Human Nutrition, University of Pavia, Via Agostino Bassi, 21, 27100 Pavia, Italy; benedetta.raspini@unipv.it (B.R.); m.morelli@bodyness.it (M.M.); hellas.cena@unipv.it (H.C.); 2 Developmental Psychiatry Unit, IRCCS Stella Maris Foundation, Viale del Tirreno 331, 56128 Pisa, Italy; m.prosperi@fsm.unipi.it (M.P.); e.santocchi@fsm.unipi.it (E.S.); r.tancredi@fsm.unipi.it (R.T.); f.muratori@fsm.unipi.it (F.M.); 3 Department of Clinical and Experimental Medicine, University of Pisa, Via Savi 10, 56126 Pisa, Italy; 4 Institute of Clinical Physiology, National Research Council, Via Moruzzi 1, 56124 Pisa, Italy; letizia.guiducci@ifc.cnr.it (L.G.); morales@ifc.cnr.it (M.A.M.); 5 Department of Pediatrics, Massachusetts General Hospital for Children, 55 Fruit Street, Boston, MA 02114, USA; MSIMIONE@mgh.harvard.edu; 6 Division of Gastroenterology and Nutrition, Massachusetts General Hospital for Children, 55 Fruit Street, Boston, MA 02114, USA; LFIECHTNER@partners.org; 7 Division of General Academic Pediatrics, Department of Pediatrics, Massachusetts General Hospital for Children, 55 Fruit Street, Boston, MA 02114, USA; 8 Clinical Nutrition and Dietetics Service, Unit of Internal Medicine and Endocrinology, ICS Maugeri IRCCS, Via S. Maugeri 10, 27100 Pavia, Italy

**Keywords:** eating habits, pediatrics, obesity, autism spectrum disorder, BMI z-score, food selectivity

## Abstract

Atypical eating habits are more common in children with autism spectrum disorders (ASD) than typically developing (TD) peers. Feeding problems may lead to the double burden of specific nutrient deficiencies and excessive weight gain, with a consequent increase in obesity prevalence. The dietary intake of Italian preschoolers with ASD compared to their TD peers and the impact of their dietary choices on their weight status and relationship to food selectivity (FS) were investigated. Dietary patterns and their associations with body mass index (BMI) were evaluated in 65 children with ASD and 82 peers with TD aged 1.3–6.4 years. Eating habits were assessed with a modified version of a parent-rated semi-quantitative Food Frequency Questionnaire. Moreover, the prevalence of FS and possible links with dietary patterns and BMI were investigated in the ASD group. Children with ASD consumed significantly higher amounts of simple sugars, processed and ultra-processed carbohydrates, both low- and high-fat animal proteins, and lower amounts of vegetables and fruits compared to peers with TD. The obesity rate was 1.5% in children with TD and more than fourfold (6.2%) in children with ASD, although the difference between groups was not statistically significant. FS was significantly more frequent in children with ASD than in peers with TD. Children with ASD and FS showed significantly lower annual intakes of vegetable proteins and fiber (considered essential nutrients for a healthy diet) than children with ASD without FS. Our results showed that children with ASD showed different dietary habits than those with TD, with the higher consumption of energy-dense foods and lower amounts of food-sourced fibers, which could place them at increased risk to develop overweight, obesity, and micronutrient deficiencies later in life.

## 1. Introduction

Autism spectrum disorders (ASD) are neurodevelopmental disorders with a prevalence rate of 1.15% in Italy [1] characterized by social and communicative deficits along with repetitive or stereotypic behavioral patterns [2]. Atypical eating behaviors and feeding problems (FPs) are more common among children with ASD than their siblings or children with typical development [3]. In particular, FPs are approximately five times more frequent in children with ASD compared to typically developing peers [4], and food selectivity (FS) is frequently reported as a common cause of FPs in this population [5]. FPs in ASD may be related to oral motor difficulties or sensory sensitivities in particular for texture, taste, and smell, possibly leading to high food refusal [6,7]. Moreover, frequently undiagnosed organic or functional GI disorders and allergies in this population might play roles in food choices and food refusal [8,9,10]. Indeed, critical dietary behaviors including food dislike, behavioral rigidity, reduced interest in mealtime, physical discomfort, and FS may influence eating habits and thus affect the nutritional status of individuals with ASD [4,11].

In light of this, a growing body of research has considered dietary patterns of children with ASD, revealing a high intake of energy-dense food (e.g., sugar-sweetened beverages and snacks) and low fruit and vegetable consumption compared to typically developing peers in the majority of cases [12]. These findings could justify the frequently reported inadequate supply of essential nutrients (e.g., vitamins and minerals) and overconsumption of energy-dense foods in children with ASD. Specific nutritional deficiencies and excessive weight gain could result, which may lead to increased obesity rates [13]. In particular, it is estimated that 30.4% of children with ASD are affected by overweight or obesity versus 23.6% of children without ASD, according to the National Survey of Children’s Health in the United States [14]. In a similar vein, a recent systematic review and meta-analysis reported a pooled prevalence of combined overweight and obesity of 37.0% in children with ASD, which was significantly greater than the prevalence of overweight and obesity for age and sex in children and adolescents aged 2–19 years in the United States (i.e., 33.4%) [15]. The aforementioned prevalence of overweight and obesity in children with ASD was also greater than the overall prevalence of overweight and obesity among 5–17-year-old children and adolescents in Italy (i.e., 27.0%) [15].

Researchers recommend health professionals to provide guidance regarding the prevention and specific management of obesity in children with ASD [16]. The United States Preventive Services Task Force suggests that providers screen children for obesity starting from the age of six and offer intensive behavioral interventions to support changes in weight status [17,18,19]. “Expert Recommendations” for weight management in primary care for children with ASD advocate the prevention of obesity much earlier, beginning at two years of age [16].

There is a growing consensus regarding the necessity to monitor nutritional status in individuals with ASD, beginning at birth [20]. Special attention must be paid to the first years of life, monitoring adiposity rebound (AR), defined as the increase in body mass index (BMI) that occurs after reaching a nadir in infancy, usually by around 5–6 years [21,22]. Epidemiologic studies have shown that an early age of AR, known as early adiposity rebound (EAR), is associated with an increased risk for later obesity [21,23].

Considering the various feeding difficulties that children with ASD face and given the consequent risks of nutrient imbalance, the aims of this study was to evaluate: (i) the dietary intake of Italian preschoolers with ASD compared to that of typically developing peers and (ii) the impact of dietary intake on weight status while considering FS.

## 2. Methods

### 2.1. Participants

A total of 147 preschoolers, 65 with ASD and 82 typically developing peers, were enrolled in this study from June 2015 to July 2016.

ASD group included 57 males and 8 females. All of them received a diagnosis of ASD according to DSM-5 [2] criteria at the ASD Unit of the IRCCS Stella Maris Foundation (Pisa, Italy), a tertiary care university hospital that cares for patients from all over Italy. ASD diagnosis was performed by a multidisciplinary team including a senior child psychiatrist and an experienced clinically trained research child psychologist, and it was confirmed through the Autism Diagnostic Observation Schedule-2 (ADOS-2) [24]. ASD patients also underwent the recommended laboratory tests to rule out medical causes of ASD, including the evaluation of thyroid function, array comparative genomic hybridization, genetic analysis for Fragile X syndrome, and screening tests for inborn errors of metabolism. Celiac disease was excluded with serological screening.

The group of typically developing children included 48 males and 34 females recruited from a kindergarten in Pisa and was age-matched to the ASD group. The inclusion criteria for this group were: (a) attending kindergarten without support teachers (the Italian law provides a support teacher for children with developmental delay and disabilities); (b) no parental concern about child development, as indicated by the negative answers of the parents to the two following questions of the Child Behavior Check List 1½–5 (CBCL 1½–5; see later) [25]: ‘Does the child have any illness or disability (either physical or mental)?’ and ‘What concerns you most about the child?’; and (c) a lack of a clinical score to the Total Problems scale of the CBCL 1½–5 (Total Problems T score lower than 60, considered as normal).

Children on special diets (e.g., gluten-free diet, casein-free diet, high-protein diet, and ketogenic diet), with known food allergies, with organic gastrointestinal illnesses that could influence dietary patterns, and on drugs/medications known to impact appetite (e.g., atypical antipsychotics, mood stabilizers, anticonvulsants, and stimulants) were excluded from both groups.

Each child with ASD underwent a medical examination during which body weight was measured in light clothing without shoes. At the same time, parents were asked to complete the questionnaires described below. For typically developing children, parents reported current weight and height at the time they completed the questionnaires required for the study.

### 2.2. Anthropometric Measures

BMI was calculated for each child as weight in kilograms divided by the square of height in meters (kg/m^2^). Children’s gender- and age-specific BMI z-scores, as well as corresponding percentiles, were calculated according to the Italian cross-sectional growth charts by Cacciari et al. [26] using the SIEDP Growth Calculator 3.0 software. Overweight was defined as a BMI percentile at or above the 85th percentile, obesity was defined as BMI percentile at or above the 95th percentile, and underweight was defined as BMI percentile below the 5th percentile [27,28]. BMI data were missing for 13 out of 82 typically developing children because parents did not report the values.

### 2.3. Assessment Instruments

#### 2.3.1. Food Frequency Questionnaire (FFQ)

The semi-quantitative Food Frequency Questionnaire (FFQ) used in this study was adapted from the original one used in the “Zoom8” survey [29,30,31] funded by the Italian Health Institute for investigating the Mediterranean diet adherence in school-age children in Italy. The self-administered questionnaire did not require specific training. The parents of each enrolled child recorded their child’s usual eating habits over the last year. For each food or food group, the parents indicated the portion size usually consumed by their child and the frequency of consumption. A legend with the equivalence between portion sizes and estimated food weights, based on the guidelines of the Official Bulletin of the Tuscany Region in school catering for children [32], was provided to parents. Thus, parents had guidance regarding how to estimate portion sizes based on the known food weights usually consumed by their child. The frequency of consumption for each food was expressed as “times per day”, “times per week”, “times per month”, “times per year”, or “never”, as performed in other investigations that used the same FFQ [29,31].

Annual intake was estimated while considering each food (in total 50 items) portion size (e.g., calculating a fraction for the small and large sizes based on the weight of the medium size). The food frequency over the whole year was calculated by converting consumption rate from 5-point scales to a continuous scale of annual consumption by multiplying the reported frequency by 365/day (e.g., if the food was consumed weekly, then the annual estimated consumption was 52.17). Finally, a score was built by multiplying the equivalent of the habitually consumed portions, as expressed in units, by the annual frequency of intake as reported by the parents. Dressing’s annual consumption (e.g., butter, oil, and cream) was calculated for each child by means of a 3-point Likert scale frequency (0 never, 1 sometimes, 2 always).

We also classified foods based on Mediterranean Diet food groups and NOVA classification [33] (Table 1). Moreover, we identified healthy and unhealthy foods according to the NOVA classifications of processed and ultra-processed foods. In particular, processed foods are described as “simple products made by adding sugar, oil, salt, unprocessed or minimally processed foods or processed culinary ingredients”. Typical examples of processed foods are canned or bottled vegetables, fruits, and legumes; salted or sugared nuts and seeds; salted, cured, or smoked meats; canned fish; and fruits in preservatives. Conversely, ultra-processed foods are described as industrial formulations with five or more ingredients. Besides salt, sugar, oils, and fats, ingredients of ultra-processed foods include food substances not commonly used in culinary preparations such as hydrolyzed proteins, modified starches, hydrogenated or interesterified oils, and additives (intended to imitate the sensorial qualities of unprocessed or minimally processed foods and their culinary preparations or to disguise undesirable qualities of the final product) such as colorants, flavorings, non-sugar sweeteners, emulsifiers, humectants, sequestrants, and firming, bulking, de-foaming, anticaking, and glazing agents [33,34,35]. Examples of typical ultra-processed products are carbonated drinks; sweet or savory packaged snacks; ice-cream, chocolate, and candies (confectionery); mass-produced packaged breads and buns; margarines and spreads; cookies (biscuits), pastries, cakes, and cake mixes; breakfast ‘cereals’, ‘cereal’, and ‘energy’ bars; ‘energy’ drinks; milk drinks, ‘fruit’ yogurts and ‘fruit’ drinks; cocoa drinks; meat and chicken extracts and ‘instant’ sauces; infant formulas, follow-on milks, and other baby products; ‘health’ and ‘slimming’ products such as powdered or ‘fortified’ meal and dish substitutes; many ready-to-heat products including pre-prepared pies, pasta dishes, and pizza dishes; poultry and fish ‘nuggets’ and ‘sticks’, sausages, burgers, hot dogs, and other reconstituted meat products; and powdered and packaged ‘instant’ soups, noodles, and desserts.

#### 2.3.2. Child Behavior Check List 1½–5

The Italian version of the CBCL 1½–5 [25] is one of the most widely used checklists to detect emotional and behavioral problems in children. It consists of 100 statements filled by parents and rated on a 3-point Likert scale (0, not true; 1, somewhat or sometimes true; 2, very true or often true).

FS was considered present if item 24 (‘Doesn’t eat well’) scored ‘2′, i.e., ‘very or often true’ and parents reported the presence of selective behavior (e.g., behaviors referred to food refusal, limited food repertoire, or a high frequency of single food intake), as in our previous investigation [8].

The answer to CBCL item 24 was available for 63 out of 65 children with ASD and 76 out of 82 typically developing peers.

### 2.4. Ethics

The study was conducted following the 1964 Declaration of Helsinki and its later amendments, as well as the International Conference on Harmonization Guidelines for Good Clinical Practice. The study was approved by the Ethical Committee of the IRCCS Stella Maris Foundation (Pisa, Italy) and the Pediatric Ethic Committee of Tuscany Region in July 2014 (Approval Number: 126/2014). Written informed consent was obtained from all parents/guardians of participants included in the study. The privacy rights of human subjects were observed.

### 2.5. Statistical Analysis

Hypotheses were specified before the data were collected. Descriptive statistics are reported as means, standard deviations, and frequencies. A chi-square test was used to determine whether there were statistically significant difference between frequencies. Student’s *t*-test was used to compare the means of normally distributed variables between children with ASD and typically developing subjects. The Pearson correlation coefficient was used to determine the strength of linear correlations. Differences between groups were regarded as statistically significant when the *p*-value was 0.05 or less.

## 3. Results

In the group of children with ASD, the mean age was 3.8 (±1.2) and 2.7 (±0.6) years, respectively, for males and females. In the group of typically developing children, the mean age was 3.7 (±1.5) and 3.7 (±1.2) years, respectively, for males and females.

In our sample, males were significantly more represented in the ASD group than in the group of typically developing children (58.5% versus 87.7%; *p* = 0.0001, respectively). Females in the ASD group were significantly younger than in the typically developing peer group (*p* = 0.03). Typically developing children were more likely to have lower BMIs compared to children with ASD (*p* = 0.045), with a concomitant lower BMI z-score trend (*p* = 0.089). Although the difference between groups was not statistically significant, the rate of obesity was 1.5% in typically developing children and more than fourfold larger (6.2%) in children with ASD. Conversely, 15.9% of typically developing peers had a BMI falling in the underweight category versus 6.2% of ASD ones, though despite nearly doubling, this difference did not reach statistical significance.

The percentage of children with FS was significantly higher (*p* = 0.025) among children with ASD (19.0%) than typically developing peers (6.6%) (see Table 2).

The dietary patterns of children with ASD and typically developing peers were quite different from each other in terms of mean portions per year for each food in the FFQ (Table 3).

Specifically, children with ASD were found to have higher levels of consumption of energy-dense foods than typically developing peers; they consumed significantly more annual portions of snacks (*p* = 0.0076), puddings (0.0008), crackers/breadsticks (0.0225), fried fish (*p* = 0.0003), French fries (*p* = 0.0196), soft drinks (*p* = 0.0178), and sweetened fruit juices (*p* = 0.0004).

Furthermore, children with ASD had higher annual intakes of low-fat milk (*p* = 0.0076), yogurt (0.0407) and red meat (*p* = 0.0404) than typically developing peers. In addition, children with ASD had lower fiber intakes than typically developing peers, including raw vegetables (*p* < 0.0001) and cereals (*p* = 0.0020). Children with ASD consumed lower amounts of canned tuna per year than typically developing peers (*p* = 0.023).

Differences in the consumption per year of cookies were also detected; the differences between groups were very close to statistical significance (*p* = 0.0510), with higher consumption in children with ASD than in typically developing peers. The consumption per year of fresh fruits and fresh fish was close to statistical significance between groups, with lower amounts in children with ASD than in typically developing peers (*p* = 0.0509 and *p* = 0.1, respectively).

No other kind of food consumption significantly differed between the two groups of children.

Expressed as “Macronutrients/fruit and vegetable intake” (see Table 1), children with ASD consumed more simple carbohydrates (*p* = 0.0005) and, in particular, simple sugars added to food (*p* = 0.0003) than typically developing peers. They also consumed more processed and ultra-processed foods (*p* = 0.0238) than typically developing individuals. Children with ASD consumed more animal proteins (*p* = 0.0060)—both low-fat (*p* = 0.0224) and high-fat ones (*p* = 0.0432)—and less vegetables and fruits (*p* = 0.0011) compared to typically developing peers (Table 4).

When examining fat consumption, children with ASD showed significant differences in the rates of annual consumption of butter (*p* = 0.001), olive oil (*p* = 0.043), margarine (*p* = 0.02), dried fruit (*p* < 0.0001), and olives (*p* = 0.001) compared to typically developing controls (Table 5). Regarding animal fat sources, only butter consumption was significantly different between the two groups. In particular, butter was “never” and “sometimes” consumed by 35.4% and 64.6% of typically developing children vs. 63.1% and 33.8% of subjects with ASD, respectively; moreover, none of the typically developing peers consumed butter “always” vs. 3.1% of those with ASD. Concerning vegetable fats, 80.5% of typically developing children consumed olive oil “never”, 15.9% consumed it “sometimes”, and 3.7% consumed it “always” vs. 72.3%, 12.3%, and 15.4%, respectively, of children with ASD; furthermore, typically developing children showed less consumption of margarine (“never”: 98.8%; “sometimes”: 1.2%; “always”: 0%) compared to children with ASD (“never”: 87.8%; “sometimes”: 10.8%; “always”: 1.5%). On the contrary, typically developing peers showed a higher consumption of dried fruit (“never”: 32.9%; “sometimes”: 65.9%; “always”: 1.2%) than children with ASD (“never”: 81.5%; “sometimes”: 15.4%; “always”: 3.1%). Significant differences were also found for olive consumption between typically developing peers (“never”: 67.1%; “sometimes”: 32.9%; “always”: 0%) and children with ASD (“never”: 90.8%; “sometimes”: 7.7%; “always”: 1.5%).

Children with ASD and FS consumed less vegetable proteins (*p* = 0.0236) and vegetables and fruits (*p* = 0.0079) than children with ASD without FS (Table 6).

Children with ASD and FS did not show different rates of annual consumption of fats from animal and vegetable sources compared to children with ASD without FS (Table 7).

Finally, no significant statistical correlation was identified between BMI z-score and macronutrients/fruit and vegetable intake in both children with ASD and typically developing peers, and no statistically significant difference was found in the BMI z-score between ASD children with and without FS.

## 4. Discussion

To our knowledge, this is the first study to evaluate the dietary intake of Italian preschoolers diagnosed with ASD, as well as the impact of dietary choices on weight status while accounting for FS in an age group ripe for intervention.

Children with ASD are often at higher risk for developing obesity (i.e., BMI-for-age ≥ 95th percentile) or overweight (i.e., BMI-for-age ≥85th percentile) than children with typical development [36,37,38,39]. Obesity is particularly challenging and its management crucial in children with ASD, since it predisposes them to several health consequences [4,39,40,41]. Therefore, it is essential to recognize modifiable risk factors for potential lifestyle changes early on.

In our sample, the prevalences of overweight and obesity in the ASD group were 23% and 6%, respectively—equivalent to more than fourfold and double, respectively, those of typically developing peers. However, these percentages were lower than in previous studies examining overweight and obesity in children with ASD [42,43,44], which reported rates between 30% and 42% for overweight and 17% and 25% for obesity.

No statistically significant differences were found between children with ASD and typically developing peers regarding rates of overweight and underweight, which was consistent with findings of a previous cross-sectional study examining children and adolescents with ASD [45]. However, the children with ASD in our sample were more likely to have higher BMIs and BMI z-scores compared to typically developing peers despite no statistically significant difference between groups (*p* = 0.089), in line with previous studies [46,47,48,49]. However, it is important to emphasize that nutritional status in children with ASD should not be exclusively assessed by means of anthropometric measurements, since subclinical nutritional inadequacies could remain under-diagnosed [4].

Evidence supports the idea that the Mediterranean dietary pattern (MDP) positively influences health and promotes the prevention of common non-communicable diseases (NCDs) [50,51]. The MDP is a dietary pattern that combines a high intake of vegetables and fruits, whole grains, legumes, nuts and seeds, and olive oil with a moderately high intake of fish and a low-to-moderate intake of dairy products, red meat, and saturated fats [52]. In our sample, children with ASD showed higher levels of consumption of energy-dense foods than typically developing peers, particularly “unhealthy food” such as snacks, puddings, crackers and breadsticks, French fries, soft drinks, and sweetened fruit juices. In contrast, children with ASD displayed a lower intake of fiber-rich foods, including raw vegetables and cereals. These findings were in accordance with other previous studies [12,46,53,54,55,56,57] reporting that children with ASD, when compared to typically developing peers, show marked preferences for sweet and savory foods and refusal for vegetables and In fruits, possibly due to their high sensitivity to bitter taste or dislike of texture [58]. By contrast, Herndon and colleagues [47] identified significantly higher consumption of fruit in children with ASD aged 4–8 years than in typically developing peers, whereas Emond et al. [46] reported that ASD toddlers consumed not only less vegetables, salads, and fresh fruit but also less sweets and fizzy drinks than typically developing peers.

Additionally, ASD preschoolers in our sample consumed a significantly higher annual amount of fried fish compared to typically developing children, as well as presented a tendency to consume less fresh fish. A previous case-control study observed that 27.3% of children with ASD never ate fish compared to 0% of typically developing peers [54]. One potential reason for the difference in types of food consumption may be related to the presence of fish bones or to texture and taste, leading caregivers to cook and prepare food to make it more palatable for children with ASD such as fried fish sticks. Furthermore, we observed that children with ASD had a higher annual consumption of other protein-rich food such as milk, yogurt, and red meat compared to typically developing peers, in line with previous data [54,55,59,60]. Regarding dairy intake, our data contrasted with previous ones. In particular, significantly less servings of dairy were found to be consumed by children with ASD than typically developing peers [47,57]. Of note, Diolordi et al. showed a higher rate of children with ASD never consuming dairy products compared to typically developing peers [54], with a statistically significant difference between the two groups for both milk and yogurt (*p* ≤ 0.005). However, none of these three investigations considered special diets (e.g., lactose-free or casein-free diets) as an exclusion criterion for the enrollment of subjects, as was performed in our sample.

When considering vegetable versus animal proteins, children with ASD showed higher intakes of the latter than typically developing peers. Other previous investigations studied the global intake of proteins, but none differentiated the protein types (i.e., animal versus vegetable) [46,47,49,57,59,61,62,63]. Currently, available data suggest that high dietary protein intake in the first two years of life and later can negatively influence the risk of obesity and associated diseases [64,65,66]. Both the quality and quantity of protein intake in infancy and childhood are of particular interest, and high protein intake, especially from animal food sources, is likely and persistently associated with adiposity up to age ten years [67,68]. The difference in the growth parameters of animal vs. vegetable proteins might be due to different amino acid compositions, since some amino acids, such as lysine and arginine, may influence insulin and growth hormone release and are thus likely to accelerate growth [67].

Similarly, carbohydrate classification allowed us to identify higher consumption of simple carbohydrates and simple sugars added to food in children with ASD than in typically developing peers. In previous studies, no statistically significant difference in carbohydrate total intake emerged between groups [46,47,49,62], although a preference for simple carbohydrates in ASD subjects had already been reported [40].

Regarding fats from animal and vegetable sources expressed as frequency per year, children with ASD showed different rates in consuming butter, olive oil, margarine, dried fruit, and olives compared to typically developing peers. Previous studies showed mixed results regarding fat consumption in children with ASD, and consistent findings are lacking [69]. Furthermore, we cannot exclude that our results could have been linked to family eating habits.

Several studies have suggested that sensory sensitivity may lead children with ASD to restrict their food choices, sticking to their preferred, tolerable, and manageable textures [5,13,70]. Food texture and taste are thought to drive food choices and impact the food acceptance of children with ASD, and children with ASD [7,56,57,70] frequently show many food dislikes, repetitive food choices, and resistance to new taste experiences [4,71,72,73]. In this study, we evaluated data related to the frequency of food consumption and how it is related to the presence or absence of FS in children with and without ASD to better understand how FS might be related to dietary patterns and affect weight status.

Our study confirmed previous findings describing a significantly higher frequency of FS in children with ASD than in typically developing peers [5,8]. However, in our sample, the percentage of FS in children with ASD was lower than previously reported [5,45,48,56,74].

Some authors have described a critical association between FS and dietary patterns with consequences on children’s weight, body composition, and nutritional status—especially for micronutrients as calcium, vitamin D, vitamin E, iron, and zinc, as well as fiber [45,75,76]. In our sample, we observed a lower consumption of legumes (expressed as vegetable proteins), fruits, and vegetables in children with ASD and FS compared to those without FS. Furthermore, we did not observe any associations of BMI or BMI z-score and macronutrients/fruit and vegetable intake annual consumption, as previously reported by other authors [4,12,56]. This finding could be partly ascribed to the age of sample composition, i.e., preschool children in whom the duration of FS may not yet have had an impact on weight and nutritional status.

Most studies investigating eating habits, food intake, and preferences are burdened by several inconsistencies and inadequacies, mainly due to different sampling designs and different methodologies; our study was no different and had limitations.

First, we did not investigate eating habits and food presentation within families. This could be particularly relevant for studies evaluating eating preferences in children with ASD and can be registered with specific mealtime behavior questionnaires [77]. Moreover, our methodology for defining dietary intake was based on a parent version of a modified FFQ. The quantity of consumed food may be biased by under- or over-reporting, and parents may not offer specific foods that are believed to be refused by the child; thus, we cannot conclude whether food choices and consumption are influenced by parent’s choices (child’s supposed or historical refusal of specific foods) or can be directly attributed to the child. In addition, we are aware that the methodological approach we used to quantify food frequency over the course of a year, i.e., the choice to convert the frequency of consumption from five-point scales to a continuous scale of annual consumption by multiplying the reported frequency of consumption to a 365 day scale could be questioned. However, this limitation is mitigated by the fact that the same procedure has been applied in recent and reliable investigations [78,79]. Future work should also capture the seasonal availability of food and changes in diet over time, as well as calculate the caloric intake. More broadly, FFQs certainly show some well-known methodological limitations [80,81], but gold-standard techniques for an objective measurement of food intake (e.g., biomarkers or doubly labelled water) are not recommended in this young and vulnerable population of ASD individuals. In this framework, the use of ad-hoc software to calculate caloric intake could greatly expand the significance of the results in the ASD population.

Secondly, we have not assessed sensory profile and oro-motor skills of participants that may influence their feeding [6,82].

In addition, we did not evaluate physical activity levels that may have affected the total energy expenditure of the enrolled subjects [12]. Finally, it should be noted that we did not use a specific tool to evaluate FS, and this may have caused some false negatives: the answer “somewhat or sometimes true” was considered equal to “not true”, taking for granted that light–moderate feeding problems were absent. Indeed, parents’ perception of their child’s feeding problem is subjective and could lead to the over- or underestimation of FPs [83].

In our sample, children with ASD and typically developing peers were not completely matched for sex and age because males were significantly more represented in the ASD group than in typically developing group, in line with the male-to-female ASD ratio reported in the literature [84]. Additionally, the mean age of ASD females was lower than that of typically developing peers. Therefore, the lack of matching between cases and controls in the abovementioned demographic aspects could have influenced dietary patterns and food choices. Moreover, height and weight were measured in the ASD group, but they were obtained from parents’ reports in the typically developing peer group, potentially leading to the misclassification of typically developing children.

Furthermore, the data collection performed between June 2015 and July 2016 represents a limitation considering that a long time has passed and changes in the nutritional offer may have occurred. Finally, our relatively small sample size of ASD children may limit the generalization of the results to the broader target population. In this context, we cannot rule out that the results we observed may not be sufficiently powered for detecting differences between experimental and control groups, leading to a type II error.

In conclusion, we found that ASD preschoolers consumed more energy-dense foods and less fruit and vegetables when compared to typically developing peers. Despite these dietary intake differences, we did not find any statistically statistical differences between dietary patterns and BMI z-scores. Therefore, we hypothesize that children with ASD may be predisposed to a little-varied diet characterized by energy-dense dietary patterns that are low in fibers, placing them at increased risk for micronutrient deficiencies and overweight and obesity later in life. Although several studies have investigated dietary patterns in children with ASD, we are still far from a complete understanding of FPs in children with ASD and therefore from tailored treatments. Further longitudinal studies with larger sample sizes would be more appropriate to evaluate the potential correlation between diet and weight status in this population in order to better understand whether alterations in dietary patterns persist into childhood and adolescence, putting children with ASD at increased risk for overweight/obesity and chronic diseases later in life. Nutritional intervention and counseling should play a pivotal role in the diversification of dietary patterns, thus supporting caregivers in their feeding activity in order to achieve nutrient intake adequacy and health status during growth. These results once again suggest the importance of early screening and management for feeding difficulties in children with ASD associated with multidisciplinary integrated care. These practices should include nutritional assessment with periodic growth follow-up (such as BMI and EAR) and the use of nutritional tools such as food diaries filled by parents and checked by pediatricians with the final aim of improving quality of life for children and their families [85].

Moreover, bodyweight is determined by energy balance, which is influenced by several behavioral, environmental, and biological factors such as food selectivity, family meals, physical activity, metabolic disorders, and genetics [36]. Considering the complex etiology of ASD and obesity, risk factors specifically associated with one condition or another are difficult to identify, but it is still essential to recognize that those risk factors for obesity or overweight are increased in children with ASD [16,36]. Therefore, a better understanding of the individual risk factors is crucial in the prevention and treatment of unhealthy excessive weight gain in children with ASD. More efforts must be made to develop early intervention strategies, personalized approaches for weight management including lifestyle and dietary recommendations, and medical therapies to avoid future deteriorations of health and quality of life.

## Figures and Tables

**Table 1:** Food groups according to Mediterranean dietary pattern and NOVA classification.

	Healthy Foods	Unhealthy Foods
Complex Carbohydrates	Bread, rice/pasta, soup, other cereals, cornmeal mush, couscous, potatoes	Focaccia, pizza, crackers/breadsticks, French fries, potato chips
Simple Carbohydrates	Honey	Shakes, puddings, stuffed pastries, not stuffed desserts, cookies, snacks, candies, ice cream, sugar, fruit juices, soft drinks, light drinks
Animal Proteins	Grated cheese, hard cheese, fresh cheese, eggs, white meat, fresh fish, whole milk, low-fat milk, skimmed milk, other milk (rice, soy, etc.), yogurt, skimmed yogurt	Melted cheese, red meat, cold cuts, fried fish, canned tuna
Vegetable Proteins	Legumes	
Fat from animal sources		Butter, cream, bacon, lard
Fat from vegetable sources	Extra virgin olive oil, olive oil, seed oil	Dried fruit, olives, margarine
Vegetables and fruits (food rich in fiber)	Fresh fruits, cooked vegetables, raw vegetables, fruit salad, fresh-squeezed juice	

**Table 2:** Participant characteristics of children with autism spectrum disorder and typically developing children.

	TD Children (*n* = 82)	Children with ASD (*n* = 65)	*p*-Value
Age, years Total, mean (SD) [min–max]	3.7 (1.3) [1.3–6.1]	3.6 (1.2) [1.6–6.4]	ns
Males, mean (SD) [min–max]	3.7 (1.5) [1.3–6.1]	3.8 (1.2) [1.7–6.4]	ns
Females, mean (SD) [min–max]	3.7 (1.2) [1.7–5.9]	2.7 (0.6) [1.6–3.6]	0.03
Sex, male (%)	58.5	87.7	0.0001
Underweight, <5th percentile BMI (%)	15.9	6.2	ns
Overweight, ≥85th percentile BMI (%)	11.6	23.1	ns
Obese, >95th percentile BMI (%)	1.5	6.2	ns
BMI, mean (SD)	15.9 (1.7)	16.6 (2.0)	0.045
BMI z-score, mean (SD)	−0.2 (1.3)	0.2 (1.0)	ns (0.089)
Food Selectivity (%)	6.6	19.0	0.025

ASD: autism spectrum disorder; BMI: body mass index; ns: not significant; SD: standard deviation; TD: typically developing.

**Table 3:** The annual food consumption of typically developing children vs. peers with ASD. Mean values are expressed in portions/year, and foods are sorted by macronutrients/fruit, vegetable intake, and eaten portions.

		TD Children (*n* = 82)	Children with ASD (*n* = 65)	ANOVA
Macronutrients/Fruit and Vegetable Intake	Food	Mean (SD) p/y	Mean (SD) p/y	*p*-Value
Complex Carbohydrates	Bread	471.14 (325.85)	443.12 (401.59)	ns
	Rice/pasta	326.24 (154.87)	392.84 (408.25)	ns
	Crackers, breadsticks	224.62 (386.92)	443.85 (743.73)	0.0225
	Soup	154.10 (127.00)	179.79 (198.83)	ns
	Focaccia	108.22 (186.48)	109.16 (197.27)	ns
	Potatoes	92.84 (88.22)	94.18 (150.17)	ns
	Pizza	66.38 (92.30)	73.74 (100.57)	ns
	Potato chips	62.61 (18.58)	28.82 (69.82)	ns
	Other cereals	37.02 (57.26)	12.20 (30.83)	0.0020
	French fries	28.60 (29.85)	59.68 (114.55)	0.0196
	Cornmeal mush	21.08 (59.48)	11.81 (54.29)	ns
	Couscous	9.20 (41.96)	0.22 (1.79)	ns
Simple Carbohydrates	Cookies	1112.55 (1074.88)	1656.99 (2197.18)	ns (0.0510)
	Snacks	192.98 (251.12)	338.64 (397.44)	0.0076
	Candies	131.76 (227.72)	299.98 (766.20)	ns
	Fruit juices	101.83 (135.40)	222.39 (261.72)	0.0004
	Honey	100.00 (173.47)	72.97 (169.74)	ns
	Sugar	81.25 (227.47)	123.54 (399.20)	ns
	Ice cream	74.68 (92.39)	85.52 (133.30)	ns
	Not stuffed desserts	65.06 (98.01)	69.88 (135.48)	ns
	Stuffed pastries	32.18 (72.65)	45.31 (102.00)	ns
	Soft drinks	23.28 (52.09)	83.21 (218.81)	0.0178
	Puddings	16.36 (51.72)	70.26 (129.75)	0.0008
	Shakes	4.30 (19.36)	28.63 (132.39)	ns
	Light drinks	0.00 (0.00)	0.185 (1.49)	ns
Animal Proteins	Whole milk	805.85 (1436.85)	600.91 (1172.27)	ns
	Low fat milk	342.16 (710.91)	842.31 (1469.84)	0.0076
	Grated cheese	310.09 (232.08)	336.20 (406.50)	ns
	White meat	149.76 (97.03)	152.29 (154.04)	ns
	Yogurt	149.13 (202.96)	355.16 (875.12)	0.0407
	Fresh fish	107.12 (171.03)	68.08 (94.38)	ns (0.1)
	Red meat	106.66 (87.90)	146.05 (141.54)	0.0404
	Other milk (rice, soy, etc.)	103.16 (522.89)	219.00 (805.38)	ns
	Hard cheese	92.64 (119.61)	138.80 (228.11)	ns
	Fresh cheese	89.55 (85.24)	101.50 (133.78)	ns
	Cold cuts	88.99 (89.19)	76.37 (108.89)	ns
	Eggs	75.82 (45.33)	102.66 (157.70)	ns
	Melted cheese	64.29 (160.72)	187.57 (692.32)	ns
	Skimmed milk	35.68 (253.97)	0.00 (0.00)	ns
	Skimmed yogurt	34.69 (77.08)	92.56 (421.41)	ns
	Fried fish	28.85 (46.97)	76.65 (102.94)	0.0003
	Canned tuna	21.89 (47.69)	7.11 (22.78)	0.023
Vegetable Proteins	Legumes	77.26 (102.89)	76.67 (99.97)	ns
Fat from vegetable sources	Olives	105.75 (275.67)	67.94 (455.45)	ns
	Dried fruit	28.04 (48.45)	16.60 (66.06)	ns
Vegetables and fruits (food rich in fiber)	Fresh fruits	625.85 (390.02)	487.75 (460.31)	ns (0.0509)
	Cooked vegetables	155.20 (134.44)	130.27 (176.11)	ns
	Raw vegetables	147.06 (164.67)	44.77 (83.92)	<0.0001
	Fruit salad	131.22 (235.85)	64.78 (174.74)	ns
	Fresh-squeezed juice	119.51 (182.16)	103.74 (169.73)	ns

ASD: autism spectrum disorder; TD: typically developing; ns: not significant; p/y: portions/year. *p*-values were derived from Mann–Whitney tests of annual food consumption in cases and controls.

**Table 4:** Comparison of annual intake of macronutrients/fruit and vegetable intake between children with ASD and typically developing children. Mean values are expressed in portions/year.

Macronutrients/Fruit and Vegetable Intake	TD Children (*n* = 82)	Children with ASD (*n* = 65)	*p*-Value
	Mean (SD) p/y	Mean (SD) p/y	
Complex Carbohydrates	1558.02 (642.28)	1849.42 (1143.90)	ns
Whole grain food	1111.62 (439.77)	1134.16 (623.02)	ns
Processed and ultra-processed food	446.40 (453.58)	715.26 (937.12)	0.0238
Simple Carbohydrates	1936.23 (1325.42)	3096.86 (2548.44)	0.0005
Simple sugars naturally found in food	100.64 (173.19)	72.97 (169.74)	ns
Simple sugars added to food	1836.229 (1279.38)	3023.89 (2514.22)	0.0003
Animal Proteins	2606.33 (1560.93)	3503.22 (2326.00)	0.0060
Low-fat	2295.65 (1548.08)	3009.46 (2196.79)	0.0224
High-fat	310.68 (272.29)	493.76 (753.63)	0.0432
Vegetable Proteins	77.26 (102.89)	76.67 (99.97)	ns
Vegetables and fruits (food rich in fiber)	1178.84 (658.04)	831.32 (594.09)	0.0011

ASD: autism spectrum disorder; *n*: number; ns: not significant; SD Standard Deviation; TD: typically developing; p/y: portions/year.

**Table 5:** Comparison of the rates of annual intake of animal and vegetable fats in each group between children with ASD and typically developing children.

Macronutrients	TD Children (*n* = 82)			Children with ASD (*n* = 65)			*p*-Value
		%			%		
Fats from animal sources	Never	Sometimes	Always	Never	Sometimes	Always	
Butter	35.4	64.6	0	63.1	33.8	3.1	0.001
Cream	64.6	35.4	0	75.4	23.1	1.5	ns
Bacon	84.1	15.9	0	90.8	7.7	1.5	ns
Lard	100	0	0	96.9	3.1	0	ns
Fats from vegetable sources	Never	Sometimes	Always	Never	Sometimes	Always	
Extra virgin olive oil	0	3.7	96.3	1.5	10.8	87.8	ns
Olive oil	80.5	15.9	3.7	72.3	12.3	15.4	0.043
Seed oil	56.1	43.9	0	70.8	27.7	1.5	ns (0.078)
Margarine	98.8	1.2	0	87.8	10.8	1.5	0.02
Dried fruit	32.9	65.9	1.2	81.5	15.4	3.1	<0.0001
Olives	67.1	32.9	0	90.8	7.7	1.5	0.001

ASD: autism spectrum disorder; ns: not significant; SD: standard deviation; TD: typically developing. The analyses of macronutrients/fruit and vegetable intake were performed by dividing the ASD sample into two subgroups on the basis of the presence or absence of FS.

**Table 6:** Comparison of annual intake of macronutrients/fruit and vegetable intake between children with ASD with and without FS. Mean values are expressed in portions/year.

Macronutrients/Fruit and Vegetable Intake	ASD Children with FS (*n* = 12)	ASD Children without FS (*n* = 51)	*p*-Value
	Mean (SD) p/y	Mean (SD) p/y	
Complex Carbohydrates	1743.89 (1764.63)	1894.87 (984.59)	ns
Whole grain food	892.20 (678.59)	1200.96 (611.21)	ns
Processed and ultra-processed food	851.69 (1743.45)	693.92 (667.75)	ns
Simple Carbohydrates	2657.62 (2328.64)	3241.94 (2645.27)	ns
Simple sugars naturally found in food	73.88 (211.47)	75.62 (163.77)	ns
Simple sugars added to food	2583.74 (2176.15)	3166.32 (2633.13)	ns
Animal Proteins	3333.80 (1493.99)	3565.75 (2520.19)	ns
Low-fat	2938.87 (1553.76)	3052.27 (2354.46)	ns
High-fat	394.94 (310.18)	513.47 (837.16)	ns
Vegetable Proteins	17.99 (32.51)	90.41 (106.29)	0.0236
Vegetables and fruits (food rich in fiber)	431.15 (378.40)	934.94 (606.32)	0.0079

ASD: autism spectrum disorder; FS: food selectivity; ns: not significant, p/y: portions/year.

**Table 7:** Comparison of the rates of annual intake of animal and vegetable fats in each group between ASD children with and without FS.

Macronutrients	ASD Children with FS (*n* = 12)			ASD Children without FS (*n* = 51)			*p*-Value
		%			%		
Fats from animal sources	Never	Sometimes	Always	Never	Sometimes	Always	
Butter	58.3	41.7	0	62.7	33.3	3.9	ns
Cream	58.3	41.7	0	78.4	19.6	2.0	ns
Bacon	91.7	8.3	0	90.2	7.8	2.0	ns
Lard	100	0	0	96.1	3.9	0	ns
Fats from vegetable sources	Never	Sometimes	Always	Never	Sometimes	Always	
Extra virgin olive oil	0	0	100	2.0	13.7	84.3	ns
Olive oil	75.0	25.0	0	70.6	9.8	19.6	ns
Seed oil	66.7	33.3	0	72.5	25.5	2.0	ns
Margarine	91.7	8.3	0	88.2	9.8	2.0	ns
Dried fruit	91.7	8.3	0	78.4	17.6	3.9	ns
Olives	83.3	16.7	0	92.2	5.9	2.0	ns

ASD: autism spectrum disorder; FS: food selectivity; *n*: number.

## References

[1] Narzisi A., Posada M., Barbieri F., Chericoni N., Ciuffolini D., Pinzino M., Romano R., Scattoni M.L., Tancredi R., Calderoni S. (2018). Prevalence of Autism Spectrum Disorder in a large Italian catchment area: A school-based population study within the ASDEU project. Epidemiol. Psychiatr. Sci..

[2] American Psychiatric Association (2013). Diagnostic and Statistical Manual of Mental Disorders.

[3] Ledford J.R., Whiteside E., Severini K.E. (2018). A systematic review of interventions for feeding-related behaviors for individuals with autism spectrum disorders. Res. Autism Spectr. Disord..

[4] Sharp W.G., Berry R.C., McCracken C., Nuhu N.N., Marvel E., Saulnier C.A., Klin A., Jones W., Jaquess D.L. (2013). Feeding Problems and Nutrient Intake in Children with Autism Spectrum Disorders: A Meta-analysis and Comprehensive Review of the Literature. J. Autism Dev. Disord..

[5] Mari-Bauset S., Zazpe I., Mari-Sanchis A., Llopis-Gonzalez A., Morales-Suarez-Varela M. (2014). Food selectivity in autism spectrum disorders: A systematic review. J. Child Neurol..

[6] Nadon G., Feldman D., Gisel E. (2013). Feeding Issues Associated with the Autism Spectrum Disorders. Recent Advances in Autism Spectrum Disorders-Volume I.

[7] Hubbard K.L., Anderson S.E., Curtin C., Must A., Bandini L.G. (2014). A comparison of food refusal related to characteristics of food in children with autism spectrum disorder and typically developing children. J. Acad. Nutr. Diet..

[8] Prosperi M., Santocchi E., Balboni G., Narzisi A., Bozza M., Fulceri F., Apicella F., Igliozzi R., Cosenza A., Tancredi R. (2017). Behavioral Phenotype of ASD Preschoolers with Gastrointestinal Symptoms or Food Selectivity. J. Autism Dev. Disord..

[9] Calderoni S., Santocchi E., Del Bianco T., Brunori E., Caponi L., Paolicchi A., Fulceri F., Prosperi M., Narzisi A., Cosenza A. (2016). Serological screening for Celiac Disease in 382 pre-schoolers with Autism Spectrum Disorder. Ital. J. Pediatr..

[10] Vissoker R.E., Latzer Y., Gal E. (2015). Eating and feeding problems and gastrointestinal dysfunction in Autism Spectrum Disorders. Res. Autism Spectr. Disord..

[11] Brown C.L., Vander Schaaf E.B., Cohen G.M., Irby M.B., Skelton J.A. (2016). Association of Picky Eating and Food Neophobia with Weight: A Systematic Review. Child. Obes..

[12] Evans E.W., Must A., Anderson S.E., Curtin C., Scampini R., Maslin M., Bandini L. (2012). Dietary patterns and body mass index in children with autism and typically developing children. Res. Autism Spectr. Disord..

[13] Peretti S., Mariano M., Mazzocchetti C., Mazza M., Pino M.C., Verrotti Di Pianella A., Valenti M. (2019). Diet: The keystone of autism spectrum disorder?. Nutr. Neurosci..

[14] Curtin C., Anderson S.E., Must A., Bandini L. (2010). The prevalence of obesity in children with autism: A secondary data analysis using nationally representative data from the National Survey of Children’s Health. BMC Pediatr..

[15] Kahathuduwa C.N., West B.D., Blume J., Dharavath N., Moustaid-Moussa N., Mastergeorge A. (2019). The risk of overweight and obesity in children with autism spectrum disorders: A systematic review and meta-analysis. Obes. Rev..

[16] Curtin C., Hyman S.L., Boas D.D., Hassink S., Broder-Fingert S., Ptomey L.T., Gillette M.D., Fleming R.K., Must A., Bandini L.G. (2020). Weight Management in Primary Care for Children With Autism: Expert Recommendations. Pediatrics.

[17] Whitlock E.P., Williams S.B., Gold R., Smith P.R., Shipman S.A. (2005). Screening and Interventions for Childhood Overweight: A Summary of Evidence for the US Preventive Services Task Force. Pediatrics.

[18] Whitlock E.P., O’Connor E.A., Williams S.B., Beil T.L., Lutz K.W. (2010). Effectiveness of weight management interventions in children: A targeted systematic review for the USPSTF. Pediatrics.

[19] Barton M. (2010). Screening for obesity in children and adolescents: US Preventive Services Task Force recommendation statement. Pediatrics.

[20] Ranjan SNasser J.A. (2015). Nutritional status of individuals with autism spectrum disorders: Do we know enough?. Adv. Nutr..

[21] González L., Corvalán C., Pereira A., Kain J., Garmendia M.L., Uauy R. (2014). Early adiposity rebound is associated with metabolic risk in 7-year-old children. Int. J. Obes..

[22] Rolland-Cachera M.F., Deheeger M., Bellisle F., Sempé M., Guilloud-Bataille M., Patois E. (1984). Adiposity rebound in children: A simple indicator for predicting obesity. Am. J. Clin. Nutr..

[23] Kang M.J. (2018). The adiposity rebound in the 21st century children: Meaning for what?. Korean J. Pediatr..

[24] Lord C. (2012). Autism Diagnostic Observation Schedule: ADOS-2.

[25] Achenbach T.M., Rescorla L.A. (2000). Manual for the ASEBA Preschool forms and Profiles.

[26] Cacciari E., Milani S., Balsamo A., Spada E., Bona G., Cavallo L., Cerutti F., Gargantini L., Greggio N., Tonini G. (2006). Italian cross-sectional growth charts for height, weight and BMI (2 to 20 yr). J. Endocrinol. Investig..

[27] Barlow S.E. (2007). Expert committee recommendations regarding the prevention, assessment, and treatment of child and adolescent overweight and obesity: Summary report. Pediatrics.

[28] Ogden C.L., Flegal K.M. (2010). Changes in Terminology for Childhood Overweight and Obesity.

[29] Martone D., Roccaldo R., Censi L., Toti E., Catasta G., D’Addesa D., Carletti C. (2013). Food consumption and nutrient intake in Italian school children: Results of the ZOOM8 study. Int. J. Food Sci. Nutr..

[30] Censi L. (2012). Studio ZOOM8: L’alimentazione e L’attività Fisica dei Bambini Della Scuola Primaria.

[31] Roccaldo R., Censi L., D’Addezio L., Toti E., Martone D., D’Addesa D., Cernigliaro A., Group Z.S., Censi L., D’Addesa D. (2014). Adherence to the Mediterranean diet in Italian school children (The ZOOM8 Study). Int. J. Food Sci. Nutr..

[32] Tuscany R. (2011). Regional Guidelines for School Catering.

[33] Monteiro C.A., Cannon G., Levy R., Moubarac J.-C., Jaime P., Martins A.P., Canella D., Louzada M., Parra D. (2016). NOVA. The star shines bright. World Nutr..

[34] Gibney M.J. (2018). Ultra-processed foods: Definitions and policy issues. Curr. Dev. Nutr..

[35] Moubarac J.-C., Batal M., Louzada M., Steele E.M., Monteiro C. (2017). Consumption of ultra-processed foods predicts diet quality in Canada. Appetite.

[36] Dhaliwal K.K., Orsso C.E., Richard C., Haqq A.M., Zwaigenbaum L. (2019). Risk factors for unhealthy weight gain and obesity among children with autism spectrum disorder. Int. J. Mol. Sci..

[37] Hyman S.L., Stewart P.A., Schmidt B., Cain U., Lemcke N., Foley J.T., Peck R., Clemons T., Reynolds A., Johnson C. (2012). Nutrient intake from food in children with Autism. Pediatrics.

[38] Zheng Z., Zhang L., Li S., Zhao F., Wang Y., Huang L., Huang J., Zou R., Qu Y., Mu D. (2017). Association among obesity, overweight and autism spectrum disorder: A systematic review and meta-analysis. Sci. Rep..

[39] Hill A.P., Zuckerman K.E., Fombonne E. (2015). Obesity and Autism. Pediatrics.

[40] Marshall J., Hill R.J., Ziviani J., Dodrill P. (2014). Features of feeding difficulty in children with Autism Spectrum Disorder. Int. J. Speech Lang. Pathol..

[41] Marí-Bauset S., Llopis-González A., Zazpe-García I., Marí-Sanchis A., Morales-Suárez-Varela M. (2015). Nutritional status of children with autism spectrum disorders (ASDs): A case-control study. J. Autism Dev. Disord..

[42] de Vinck-Baroody O., Shui A., Macklin E.A., Hyman S.L., Leventhal J.M., Weitzman C. (2015). Overweight and Obesity in a Sample of Children With Autism Spectrum Disorder. Acad. Pediatr..

[43] Criado K.K., Sharp W.G., McCracken C.E., De Vinck-Baroody O., Dong L., Aman M.G., McDougle C.J., McCracken J.T., Eugene Arnold L., Weitzman C. (2018). Overweight and obese status in children with autism spectrum disorder and disruptive behavior. Autism.

[44] Curtin C., Jojic M., Bandini L.G. (2014). Obesity in children with autism spectrum disorder. Harv. Rev. Psychiatry.

[45] Bandini L.G., Anderson S.E., Curtin C., Cermak S., Evans E.W., Scampini R., Maslin M., Must A. (2010). Food Selectivity in Children with Autism Spectrum Disorders and Typically Developing Children. J. Pediatr..

[46] Emond A., Emmett P., Steer C., Golding J. (2010). Feeding symptoms, dietary patterns, and growth in young children with autism spectrum disorders. Pediatrics.

[47] Herndon A.C., DiGuiseppi C., Johnson S.L., Leiferman J., Reynolds A. (2009). Does nutritional intake differ between children with autism spectrum disorders and children with typical development?. J. Autism Dev. Disord..

[48] Schmitt L., Heiss C.J., Campbell E.E. (2008). A comparison of nutrient intake and eating behaviors of boys with and without Autism. Top. Clin. Nutr..

[49] Zimmer M.H., Hart L.C., Manning-Courtney P., Murray D.S., Bing N.M., Summer S. (2012). Food variety as a predictor of nutritional status among children with Autism. J. Autism Dev. Disord..

[50] Abdelhamid A., Jennings A., Hayhoe R.P.G., Awuzudike V.E., Welch A.A. (2020). High variability of food and nutrient intake exists across the Mediterranean Dietary Pattern—A systematic review. Food Sci. Nutr..

[51] Cena HCalder P.C. (2020). Defining a Healthy Diet: Evidence for The Role of Contemporary Dietary Patterns in Health and Disease. Nutrients.

[52] Trichopoulou A., Martínez-González M.A., Tong T.Y.N., Forouhi N.G., Khandelwal S., Prabhakaran D., Mozaffarian D., de Lorgeril M. (2014). Definitions and potential health benefits of the Mediterranean diet: Views from experts around the world. BMC Med..

[53] Ahearn W.H., Castine T., Nault K., Green G. (2001). An assessment of food acceptance in children with autism or pervasive developmental disorder-not otherwise specified. J. Autism Dev. Disord..

[54] Diolordi L., del Balzo V., Bernabei P., Vitiello V., Donini L.M. (2014). Eating habits and dietary patterns in children with Autism. Eat. Weight Disord..

[55] Malhi P., Venkatesh L., Bharti B., Singhi P. (2017). Feeding Problems and Nutrient Intake in Children with and without Autism: A Comparative Study. Indian J. Pediatr..

[56] Schreck K.A., Williams K. (2006). Food preferences and factors influencing food selectivity for children with autism spectrum disorders. Res. Dev. Disabil..

[57] Schreck K.A., Williams K., Smith A.F. (2004). A comparison of eating behaviors between children with and without Autism. J. Autism Dev. Disord..

[58] Coulthard H., Blissett J. (2009). Fruit and vegetable consumption in children and their mothers. Moderating effects of child sensory sensitivity. Appetite.

[59] Levy S.E., Souders M.C., Ittenbach R.F., Giarelli E., Mulberg A.E., Pinto-Martin J.A. (2007). Relationship of dietary intake to gastrointestinal symptoms in children with autistic spectrum disorders. Biol. Psychiatry.

[60] Bowers L. (2002). An audit of referrals of children with autistic spectrum disorder to the dietetic service. J. Hum. Nutr. Diet..

[61] Cornish E. (2002). Gluten and casein free diets in autism: A study of the effects on food choice and nutrition. J. Hum. Nutr. Diet..

[62] Johnson C.R., Handen B.L., Mayer-Costa M., Sacco K. (2008). Eating habits and dietary status in young children with Autism. J. Dev. Phys. Disabil..

[63] Lockner D.W., Crowe T.K., Skipper B.J. (2008). Dietary intake and parents’ perception of mealtime behaviors in preschool-age children with autism spectrum disorder and in typically developing children. J. Am. Diet. Assoc..

[64] Koletzko B., Demmelmair H., Grote V., Prell C., Weber M. (2016). High protein intake in young children and increased weight gain and obesity risk. Am. J. Clin. Nutr..

[65] Hörnell A., Lagström H., Lande B., Thorsdottir I. (2013). Protein intake from 0 to 18 years of age and its relation to health: A systematic literature review for the 5th Nordic Nutrition Recommendations. Food Nutr. Res..

[66] Pimpin L., Jebb S.A., Johnson L., Llewellyn C., Ambrosini G.L. (2018). Sources and pattern of protein intake and risk of overweight or obesity in young UK twins. Br. J. Nutr..

[67] Braun K.V., Erler N.S., Kiefte-de Jong J.C., Jaddoe V.W., van den Hooven E.H., Franco O.H., Voortman T. (2016). Dietary Intake of Protein in Early Childhood Is Associated with Growth Trajectories between 1 and 9 Years of Age. J. Nutr..

[68] Jen V., Braun K.V.E., Karagounis L.G., Nguyen A.N., Jaddoe V.W.V., Schoufour J.D., Franco O.H., Voortman T. (2019). Longitudinal association of dietary protein intake in infancy and adiposity throughout childhood. Clin. Nutr..

[69] Marí-Bauset S., Llopis-González A., Zazpe I., Marí-Sanchis A., Suárez-Varela M.M. (2016). Fat intake in children with autism spectrum disorder in the Mediterranean region (Valencia, Spain). Nutr. Neurosci..

[70] Postorino V., Sanges V., Giovagnoli G., Fatta L.M., De Peppo L., Armando M., Vicari S., Mazzone L. (2015). Clinical differences in children with autism spectrum disorder with and without food selectivity. Appetite.

[71] Curtin C., Hubbard K., Anderson S.E., Mick E., Must A., Bandini L.G. (2015). Food selectivity, mealtime behavior problems, spousal stress, and family food choices in children with and without autism spectrum disorder. J. Autism Dev. Disord..

[72] Bandini L.G., Curtin C., Eliasziw M., Phillips S., Jay L., Maslin M., Must A. (2019). Food selectivity in a diverse sample of young children with and without intellectual disabilities. Appetite.

[73] Stafford L.D., Tsang I., López B., Severini M., Iacomini S. (2017). Autistic traits associated with food neophobia but not olfactory sensitivity. Appetite.

[74] Suarez M.A., Nelson N.W., Curtis A.B. (2014). Longitudinal follow-up of factors associated with food selectivity in children with autism spectrum disorders. Autism.

[75] Matheson BEDouglas J.M. (2017). Overweight and obesity in children with autism spectrum disorder (ASD): A critical review investigating the etiology, development, and maintenance of this Relat. J. Autism Dev. Disord..

[76] Barnhill K., Gutierrez A., Ghossainy M., Marediya Z., Marti C.N., Hewitson L. (2017). Growth status of children with autism spectrum disorder: A case–control study. J. Hum. Nutr. Diet..

[77] Poppert K.M., Patton S.R., Borner K.B., Davis A.M., Dreyer Gillette M.L. (2015). Systematic review: Mealtime behavior measures used in pediatric chronic illness populations. J. Pediatr. Psychol..

[78] Ryman T.K., Austin M.A., Hopkins S., Philip J., O’Brien D., Thummel K., Boyer B.B. (2014). Using exploratory factor analysis of FFQ data to identify dietary patterns among Yup’ik people. Public Health Nutr..

[79] Ryman T.K., Boyer B.B., Hopkins S., Philip J., O’brien D., Thummel K., Austin M.A. (2015). Characterising the reproducibility and reliability of dietary patterns among Yup’ik Alaska Native people. Br. J. Nutr..

[80] Lafay L., Mennen L., Basdevant A., Charles M., Borys J., Eschwege E., Romon M. (2000). Does energy intake underreporting involve all kinds of food or only specific food items?. Results from the Fleurbaix Laventie Ville Sante (FLVS) study. Int. J. Obes..

[81] Huybrechts I., De Backer G., De Bacquer D., Maes L., De Henauw S. (2009). Relative validity and reproducibility of a food-frequency questionnaire for estimating food intakes among Flemish preschoolers. Int. J. Environ. Res. Public Health.

[82] Nadon G., Feldman D.E., Dunn W., Gisel E. (2011). Association of sensory processing and eating problems in children with autism spectrum disorders. Autism Res. Treat..

[83] Harvey L., Bryant-Waugh R., Watkins B., Meyer C. (2015). Parental perceptions of childhood feeding problems. J. Child. Health Care.

[84] Werling DMGeschwind D.H. (2013). Sex differences in autism spectrum disorders. Curr. Opin. Neurol..

[85] Simione M., Dartley A.N., Cooper-Vince C., Martin V., Hartnick C., Taveras E.M., Fiechtner L. (2020). Family-centered Outcomes that Matter Most to Parents: A Pediatric Feeding Disorders Qualitative Study. J. Pediatr. Gastroenterol. Nutr..

